# The Role of GM130 in Nervous System Diseases

**DOI:** 10.3389/fneur.2021.743787

**Published:** 2021-10-28

**Authors:** Bei Huang, Xihong Li, Xiaoshi Zhu

**Affiliations:** ^1^Operational Management Office, West China Second University Hospital, Sichuan University, Chengdu, China; ^2^Key Laboratory of Birth Defects and Related Diseases of Women and Children (Sichuan University), Ministry of Education, Chengdu, China; ^3^Emergency Department, West China Second University Hospital, Sichuan University, Chengdu, China; ^4^Pediatric Intensive Care Unit, Sichuan Provincial People's Hospital, Chengdu, China

**Keywords:** GM130, AD, PD, ALS, MCPH, SAE, ataxia, nervous system diseases

## Abstract

Golgi matrix protein 130 (GM130) is a Golgi-shaping protein located on the cis surface of the Golgi apparatus (GA). It is one of the most studied Golgin proteins so far. Its biological functions are involved in many aspects of life processes, including mitosis, autophagy, apoptosis, cell polarity, and directed migration at the cellular level, as well as intracellular lipid and protein transport, microtubule formation and assembly, lysosome function maintenance, and glycosylation modification. Mutation inactivation or loss of expression of GM130 has been detected in patients with different diseases. GM130 plays an important role in the development of the nervous system, but the studies on it are limited. This article reviewed the current research progress of GM130 in nervous system diseases. It summarized the physiological functions of GM130 in the occurrence and development of Alzheimer's disease (AD), Parkinson's disease (PD), amyotrophic lateral sclerosis (ALS), microcephaly (MCPH), sepsis associated encephalopathy (SAE), and Ataxia, aiming to provide ideas for the further study of GM130 in nervous system disease detection and treatment.

## Introduction

As a highly dynamic organelle, Golgi apparatus (GA) acts importantly in regulating cell homeostasis. Many diseases related to endoplasmic reticulum (ER)-to-Golgi or Golgi internal transport, including virus infection, cancer, ischemic stress, various nervous system diseases, alcoholic liver injury, and so on, show serious dysfunction of Golgi structure and function (1–5).

Golgi matrix protein 130 (GM130) is the first reported matrix protein that regulates the structure of GA (6). It was first identified in the screening of novel GA-associated proteins in 1995 (6). It is encoded by the *GOLGA2* gene and is one of the most studied Golgin proteins so far. GM130 is a Golgi-shaping protein, tightly bound to Golgi membranes. Maintaining the advanced structure of GA is the most important function of GM130. In addition, it plays a key role in fusion between Golgi membranes and transport vesicles originating from ER (7), spindle assembly and cell division (8), nucleation of microtubules on the Golgi (9), as well as regulation of the compartmental organization in dendritic Golgi outposts (10).

Mutation inactivation or loss of expression of GM130 has been detected in patients with different diseases. GM130 expression is lost in patients with colorectal cancer (11) and breast cancer (11–13). However, the high expression of GM130 predicted shorter survival in patients with gastric cancer (14). Diacylglycerol acyltransferase I (DGAT1) can inhibit prostate cancer by regulating the amount of microtubule-organizing center (MTOC) and GM130 and damaging microtubule integrity (15). The appearance of high mannose N-glycans on cell surface and the Golgi localization of α-mannosidase 1A at GM130-Golgi Reassembly and Stacking Protein 65 (GRASP65) may be the markers of malignant prostate cancer cells (16). In addition, damaging GM130-GRASP65 binding leads to the degradation of GM130, resulting in GA fragmentation, and leading to acute pancreatitis in mice (17). In cells lacking α-N-acetylglucosaminidase (NAGLU), GM130 expression increased, Golgi structure expanded and elongated, and abnormal lysosome accumulated, while inhibiting the expression of GM130 could restore the pathological phenotype lacking NAGLU (18). GM130 and mammalian GA play key roles in controlling the secretion of surfactant proteins in pulmonary epithelial cells (19). Moreover, the knockout of GM130 in the nervous system could lead to progressive death of Purkinje cells in the cerebellum. The mice showed obvious dyskinesia, decreased motor balance ability, and unstable standing. In the tail suspension test, the mice would rotate violently, and then the hook reflex occurred. Some mice showed symptoms similar to cerebellar ataxia. In severe cases, they would have paralysis symptoms, and these symptoms had degenerative characteristics (20). In zebrafish, inactivation caused by GM130 mutation resulted in severe skeletal muscle dysgenesis and progressive microcephaly (MCPH). The patients with the same GM130 homozygous mutation showed MCPH, myofibrillar atrophy, hypotonia, and growth retardation, and all symptoms showed obvious degeneration (21).

GM130 acts critically in the development of nervous system, but the studies on it are limited. In this paper, we reviewed the research of GM130 in nervous system diseases such as Alzheimer's disease (AD), Parkinson's disease (PD), amyotrophic lateral sclerosis (ALS), MCPH, sepsis associated encephalopathy (SAE), and Ataxia, aiming to provide a reference for the further study of GM130 in nervous system disease detection and treatment.

## The Structure of GM130

**Figure 1:**
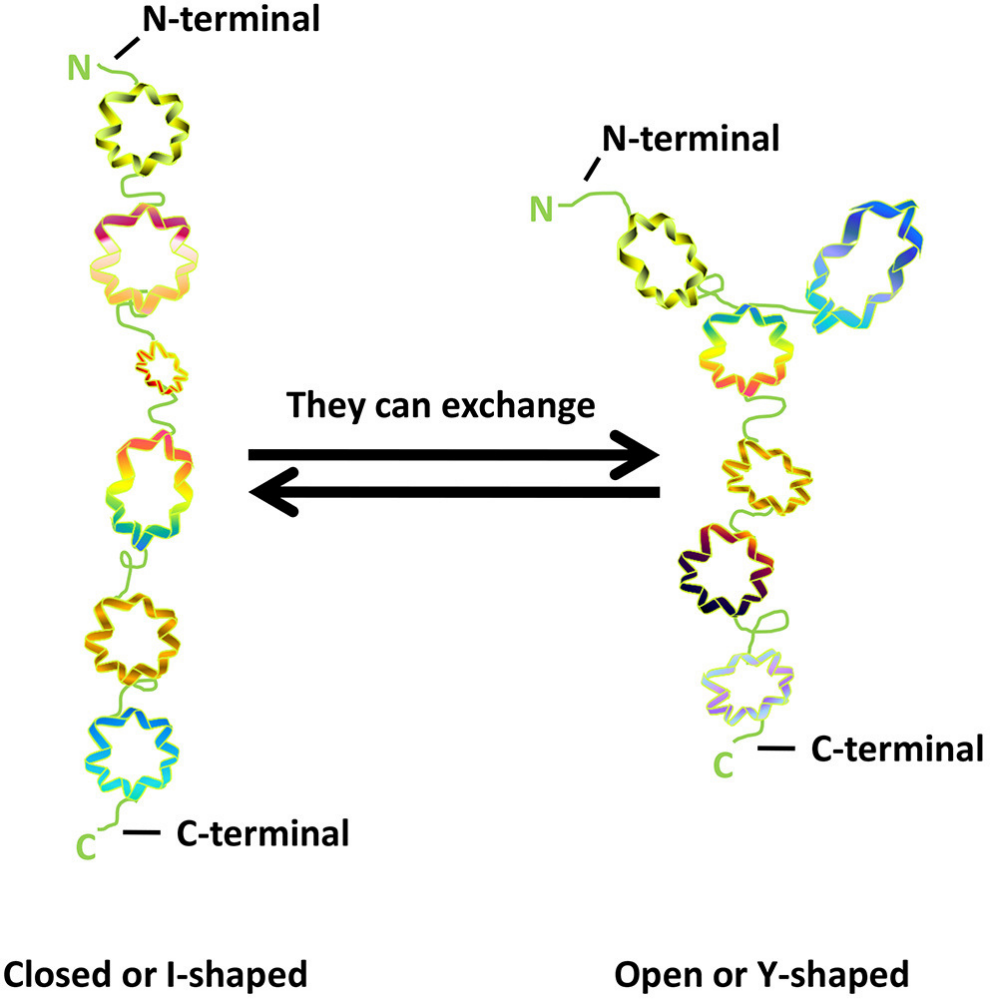
The structure of GM130.

GM130 is tightly bound to Golgi membrane, located on the cis surface of GA, and is part of the cis-Golgi matrix (6). According to the primary amino acid sequence, Nakamura et al. (6) predicted that more than 60% of the entire GM130 molecule contains coiled-coil structures and possibly exceed 90%, through the method of Lupas et al. (22). They are located in the intermediate region of GM130 molecules, allowing GM130 to have a cord-like three-dimensional structure that facilitates the capture of vesicles and the connection of GA membrane. GM130 is bound to the Golgi membrane through the C-terminal region (23). It also binds GRASP65, a peripheral Golgi membrane protein that might play a role in cisternal stacking through its C-terminal PSD95-DlgA-zo-1 (PDZ) ligand motif (24–26). The N-terminal of GM130 is positively charged and can bind to p115, another matrix protein of GA, which is used to capture vesicles in transport (27). The study of Ishida et al. (28) showed that GM130 has similar frequencies of I- and Y-shaped conformations, indicating that the N-terminal region could exchange between non-branched state (closed or I-shaped) and branched state (open or Y-shaped) (Figure 1).

## Physiological Functions of GM130 in Nervous Systems

GM130 plays a critical role in nervous system development. It has many biological functions, including its roles in maintaining the structure of GA, participating in transporting proteins and lipids, influencing mitosis, regulating migrating and polarizing cells, as well as in efficient glycosylation.

### Maintain the Structure of GA

GA is an important part of the endomembrane system. Alterations of the conventional Golgi organization are associated with different neurodegenerative diseases (29). As a matrix protein of the GA, the most noteworthy function of GM130 is to maintain the ribbon structure of GA (30, 31). The abnormal GA structure is manifested by the decrease of GM130 expression (32). An important step in Golgi ribbon biogenesis is to fully incorporate the ER-to-Golgi carriers (EGCs) into the stacks, which requires the continuous circulation of GM130 between cis-Golgi and EGCs (30). The absence of GM130 disrupted this process, resulting in the accumulation of tubular vesicle membranes, the shortening of flat ER vesicles, and the decomposition of Golgi bands (30).

**Figure 2:**
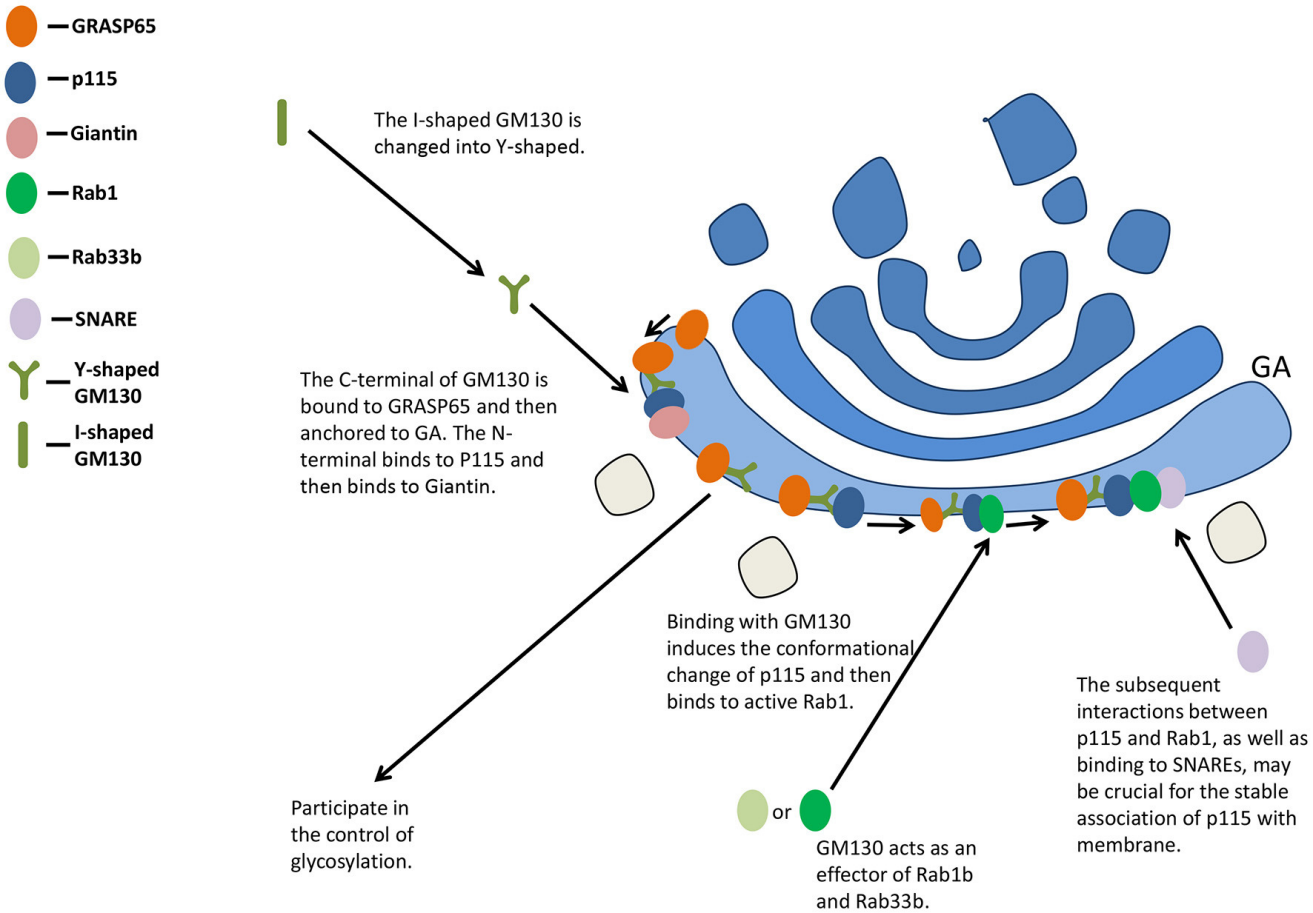
GM130 maintains the structure of GA and participates in the control of glycosylation. GA, Golgi apparatus; SNARE, soluble N-ethylmaleimide-sensitive factor attachment protein receptor.

The C-terminal of GM130 is bound to GRASP65 and then anchored to GA, while the N-terminal binds to P115 and then binds to Giantin positioned on the vesicle membrane, participating in the maintenance of the cis-face ribbon structure of GA (33, 34) (Figure 2). Binding with GM130 induces the conformational change of p115 from a self-inhibitory state to one capable of binding to active Rab1 (35). The subsequent interactions between p115 and Rab1, as well as binding to unassembled soluble N-ethylmaleimide-sensitive factor attachment protein receptors (SNAREs), may be crucial for the stable association of p115 with membrane (36). The overexpression of GM130 lacking N-terminal peptide or microinjection of N-terminal peptide of GM130 inhibits the binding of p115 to Golgi membranes (37).

Eisenberg-Lerner et al. (38) found that even the partial degradation of GM130 is enough to cause harm to GA organization. The deletion of heme oxygenase-1 (HO-1) could aggravate Golgi stress and Golgi fragmentation via decreasing the expression of GM130, Golgi-associated protein golgin A1 (Golgin 97), and mannosidase II (39). The binding of p97/VCP and 26S proteasomes to Golgi membrane or activation of Golgi stress induces GM130 degradation, causing Golgi fragmentation in turn (38). The p115-GM130 tethered complex is disrupted by GM130 phosphorylation on serine 25 (Ser-25) mediated by cyclin dependent kinase 1 (Cdk1) during mitosis, resulting in the perturbation of Golgi structure (40). At telophase mitosis, GM130 is dephosphorylated by protein phosphatase 2A (PP2A) and Golgi is reassembled to form the ribbon structure (40). Zhou et al. (41) indicated that protein arginine methyltransferase 5 (PRMT5) interacted with GM130, localized to the GA, and regulated the formation of Golgi ribbon through methylation of GM130.

### Participate in Transporting

The Golgi complex (GC) could absorb a large amount of membrane input from the ER, and the membrane input might be equal to or even exceed the surface area of Golgi stacks themselves under certain conditions (42). Such incoming membranes act as pleiomorphic EGCs. GM130 interacts with other proteins involved in membrane transport within cells. It is an effector of Rab1b and Rab33b, influencing the intra-Golgi and ER-Golgi docking and fusion *in vitro* (43, 44). P115, GRASP65, GM130, and Giantin can form complexes, which may be molecular tethers between the vesicle and the acceptor membrane before fusion (30).

**Figure 3:**
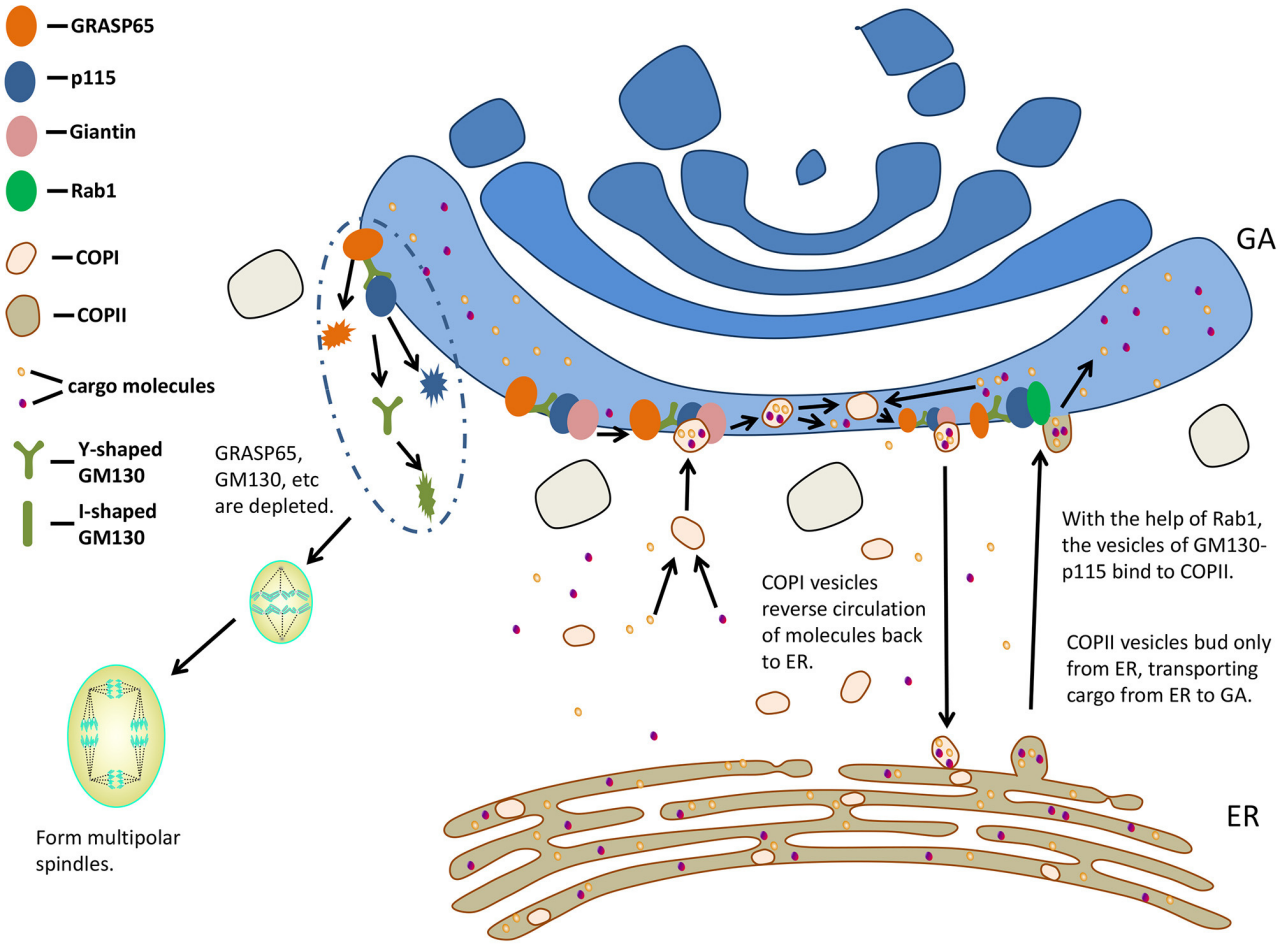
GM130 participates in transporting and influences mitosis. GA, Golgi apparatus; ER, endoplasmic reticulum; COP, coat protein.

Vesicles mediate transport along the secretory pathway (37). Two coated vesicles, coat protein (COP) I and COPII, are involved in the early part of this pathway. COPI vesicles are associated with anterograde transport of cargo molecules via Golgi stacks (45) and/or reverse circulation of molecules back to ER (46, 47), whereas COPII vesicles bud only from ER, transporting cargo from ER to GA (48, 49). GM130 was shown to be required for COPI vesicles to dock with the acceptor Golgi cisternae (50). With the help of small GTPase Rab1, the vesicles of GM130-p115 bind to the budding COPII vesicles of ER, regulating the transport from ER to the cis surface of GA (51, 52). P115, GM130, and Giantin complexes are responsible for mediating COPII vesicles to the cis surface of GA (53) (Figure 3).

Interfering with GM130 could lead to vesicular membrane accumulation and inhibition of ER-to-Golgi transport (33). The cytoplasmic domain of Human Ether-à-go-go-Related Gene (HERG) binds to GM130 (54), and the normal movement of HERG from ER to GA could be upset by the pathogenic mutation (55) in this domain. Roti et al. (54) proposed that the cytoplasmic C-terminals of HERG were involved in tethering or possibly targeting of HERG-containing vesicles in Golgi through interacting with GM130. GM130 and Giantin are required to deliver cargo proteins to the Golgi compartment containing mannosidase II (33). Golgi peripheral membrane protein GM130 and vesicle tethering factor p115 promote transporting vesicles to Golgi (50, 56). GCC88, golgin-97, and golgin-245 are three of the four golgins sharing a C-terminal GRIP. They all capture endosome to Golgi cargo, while Golgi-microtubule-associated protein of 210 kDa (GMAP-210) and GM130 capture ER to Golgi carriers (57). They may work together to surround different Golgi regions with docking sites of specific vesicle types (52). Treating Chinese hamster ovary (CHO) cells without GM130 expression at 39.5°C caused structural damage of GA, and transport from ER to GA was significantly affected (58). The disruption of p115 and GM130 tethered complexes caused increased transport vesicles and transport inhibition, suggesting that efficient transportation of cargo through Golgi requires tethering (37). Loss of function of GM130 impairs the transfer of membrane to the top of dendrites through the loss of Golgi positioning and decreased ER-to-Golgi traffic rate, probably resulting from the defects in vesicle tethering (20).

### Influence Mitosis

The Golgi membranes in mammalian cells fragment as it enters the mitotic cycle, while the fragmentation of GA is not a result of mitosis, but a key to regulating the entry of cells into mitosis (59). *In vitro* experiment of reconstructing mitotic specific fragmentation of Golgi membranes, the addition of GRASP65 prevented Golgi from fragmenting (60). The proteins localized at the Golgi (GRASP65, Sac1, and Tankyrase) are necessary for the normal function of centrosome during mitosis. When these proteins are depleted, abnormal multipolar spindles could be observed (61, 62).

GM130 regulates the binding of GRASP65 to GA, which is required for the formation of a bipolar mitotic spindle. Therefore, GM130 affects spindle formation indirectly (63). In addition, GM130 regulates the localization and stability of GRASP65 (64). The loss of GM130 resulted in centrosome abnormalities and non-function. They had many γ-tubulin-negative and Centrin2-positive foci, failing to organize microtubules during mitosis and interphase (63). A Golgi-associated complex consisting of GM130, Cdc42, the Rho GTPase, and Tuba regulates the normal centrosome morphology during interphase (65). By binding to Tuba at GA, GM130 activated a subset of Cdc42, thereby regulating centrosomal organization of unstimulated cells (65). When entering mitosis, GM130-depleted cells formed multipolar spindles. They were arrested in metaphase and then died (63). The spindle assembly factor targeting protein for xenopus kinesin-like protein 2 (TPX2) is activated by GM130 on Golgi membranes to promote the growth of astral microtubule (66). The nuclear localization signal (NLS)-like motifs of GM130 were thought to isolate Importin-α from spindle assembly factor TPX2, and then stimulated microtubule nucleation during mitosis (8). Chang et al. (67) hypothesized that the association of Importin-α with GM130 during mitosis might inhibit the interaction between GM130 and p115, resulting in the disintegration of Golgi. During early mitosis, Cdk1 phosphorylates Ser-25 residues in the GM130 NLS-like motif, and such GM130 phosphorylation is associated with mitotic Golgi disassembly (27, 40). The deletion of GM130 or the injection of GM130 antibody into cells resulted in aberrant centrosome replication and formation of multipolar spindles, leading to abnormal mitosis (67). GM130 regulated microtubule organization and might play a role in aberrant spindle and asymmetric division during oocyte maturation in mice (68).

### Regulate Cell Polarization and Migration

Cell polarity, a highly coordinated multistep cellular process, regulates multiple biological functions related to wound healing, cell migration, and cancer (11). Golgi is considered to be important in cell polarization (69, 70). The knock-out of GM130, Stk25, and liver kinase B1 (LKB1) resulted in Golgi dispersion (71), reducing its effect on cell polarity (72). Stk25 regulates polarized migration in cultured cells through its interactions with GM130 (73).

GM130 also interacts with the signaling molecule kinase YSK1 to regulate cell migration and polarity. The mammalian sterile 20 (Ste20) kinases YSK1 target GA via GM130, whose binding activates these kinases through facilitating autophosphorylation of conserved threonine within the T-loop. Interfering with the function of YSK1 disturbs perinuclear Golgi organization and cell migration (73).

The small GTPase Cdc42 is a key polarity regulator (74). A GM130-RasGRF complex was reported as a regulator of Cdc42 at GA (11). RasGRF family guanine nucleotide exchange factors are regulators of small GTPase Ras (75, 76), while RasGRF2 is a novel interaction partner for GM130 (11). Silencing GM130 could induce RasGRF to specifically inhibit the activity of small GTPase Cdc42 on GA and activate the Ras GRF-dependent Ras-extracellular signal-regulated kinase (Ras-ERK) pathway, inducing the loss of cell polarity. Golgi polarity was lost after short hairpin RNA (shRNA)-mediated depletion of GM130 in hippocampal granule cells (77). GM130 is not necessary for the initial polarization of Golgi, but it contributes to maintaining the polarized distribution of GA in Purkinje cells, probably via binding to AKAP450 and centrosome (20).

**Figure 4:**
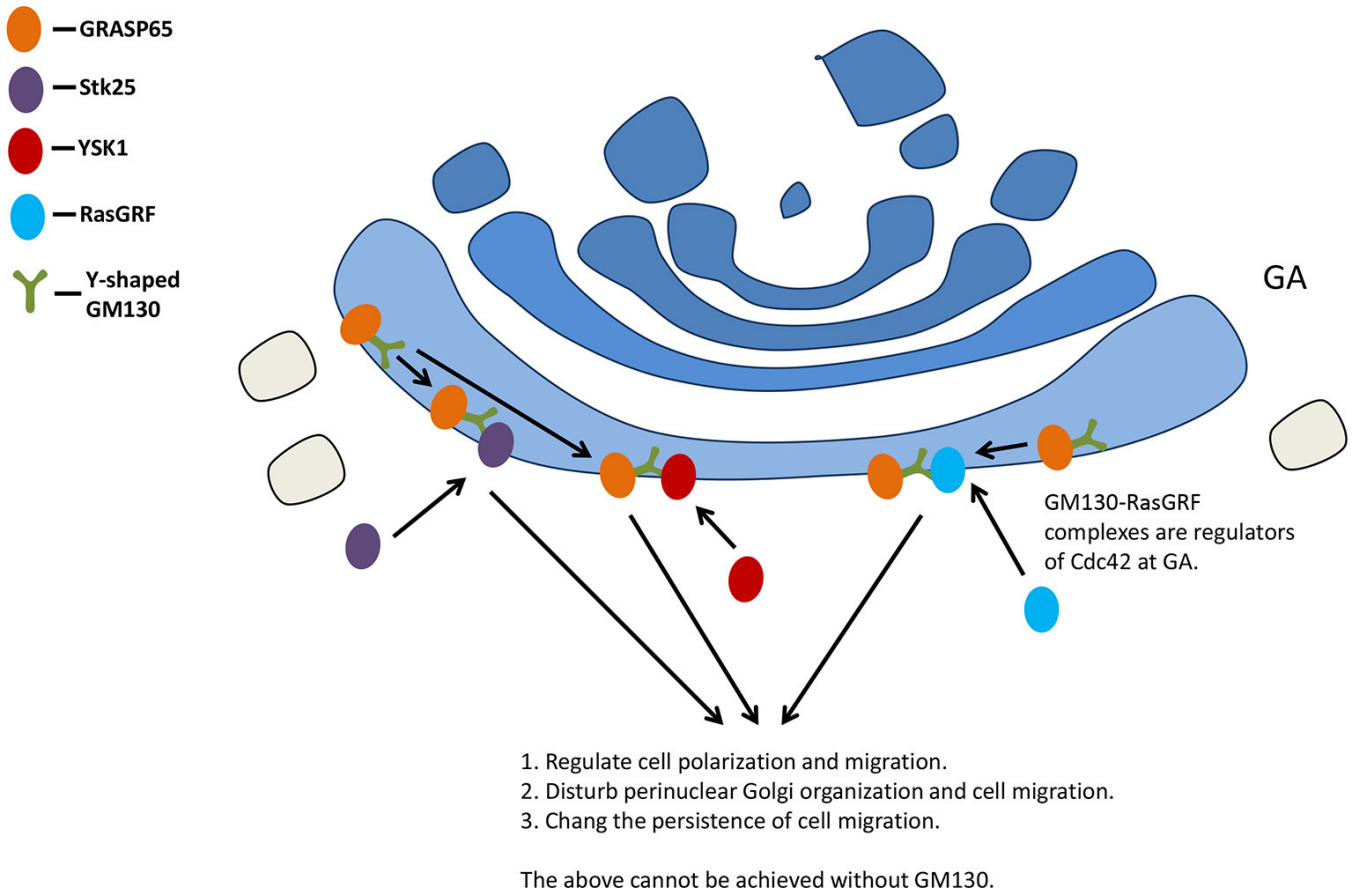
GM130 regulates cell polarization and migration. GA, Golgi apparatus.

Studies have demonstrated the effect of GM130 deletion on cell migration: the loss of GM130 inhibited directional motility and increased random cell motility at the same time (13). GM130 regulates the original polarity of cells by regulating the balance between Cdc42 and Ras signals, and changes the persistence of cell migration (13) (Figure 4).

### Participate in the Control of Glycosylation

Even if the glycosylation patterns of surface or secreted proteins changes slightly, it would induce various cellular phenomena, disrupting the homoeostasis of tissues (78, 79), while GM130 may be involved in providing a suitable glycosylation environment. The GM130-GRASP65 complex might physically connect adjacent Golgi stacks, then allow lateral membrane fusion and enzyme balance to obtain the best processing environment (64). Giantin deficiency in androgen-independent prostate cancer cells leads to Golgi targeting glycosyltransferases and α-mannosidase IA transferring from Giantin to GM130-GRASP65, and the disorder of glycosylation environment at this site would cause the complete change of downstream glycosylation pathway (80). The study of Chang et al. (81) also showed that the down-regulation of GM130 might cause glycosylation deficiency in cancer cells. The production of abnormal O-glycosylation IgA1 is a major cause of IgA nephropathy, while the down-regulation of GM130 increases IgA1 O-glycosylation deficiency. Via negatively regulating the expression of β1, 3-Gal transferase (C1GalT1), GM130 is of critical importance in IgA1 O-glycans deficiency in IgAN patients (82).

In addition, Golgi-ribbon architecture contributes to cell-type specific glycosylation patterns in mammals. Knockout of GM130 led to the absence of Golgi-ribbon formation, related to increased enzyme deviation and the defective sialylation of cell surface proteins (Table 1).

**Table 1:** The main physiological functions of GM130 in nervous system.

**References**	**Function**	**Mechanism**
Alvarez et al. (33), Zhang and Seemann (34)	Maintain the structure of GA	The C-terminal of GM130 binds to GRASP65 and is then anchored to GA, while the N-terminal binds to P115 and then binds to Giantin positioned on the vesicle membrane, participating in the maintenance of the cis-face ribbon structure of GA
Alvarez et al. (33)	Participate in transporting	Interfering with GM130 could lead to vesicular membrane accumulation and ER-to-Golgi transport inhibition
Kodani and Sütterlin (63)	Influence mitosis	The depletion of GM130 led to abnormal interphase centrosomes and nonfunctional multipolar spindles during mitosis
Preisinger et al. (73), Baschieri et al. (13)	Regulate cell polarization and migration	GM130 interacts with the signaling molecule kinase YSK1 to regulate cell migration and polarity. GM130 regulates the original polarity of cells by regulating the balance between Cdc42 and Ras signals
Puthenveedu et al. (64)	Participate in the control of glycosylation	Golgi-ribbon architecture contributes to cell-type specific glycosylation patterns in mammals and the knockout of GM130 leads to the absence of Golgi-ribbon formation

*GA, Golgi apparatus; ER, endoplasmic reticulum*.

## The Study of GM130 in Nervous System Diseases

In developing neurons, GA could serve as non-centrosome-associated outposts, being important for transporting cargo directly to the newly formed dendritic plasma membrane and local microtubule nucleation to help dendrite morphogenesis (10, 83–85). GA fragment in neurodegenerative diseases such as AD (86), PD (87), ALS (88), and spinocerebellar ataxia type 2 (SCA2) (89). GM130 is conducive to the ribbon morphology of Golgi, tethering transport vesicles to promote ER-to-Golgi traffic (30, 37), Golgi positioning and cytoskeletal regulation (7, 8), as well as the organization of neuronal Golgi outposts (10). The accumulation of vesicles, Golgi apparatus disorganization, and other alterations in GM130 function may account for neuron dysfunction and death (90). Partial loss of GM130 function in human induced pluripotent stem cells and neurons affected stem cell polarity, motility, migration, as well as neurogenesis and neuritogenesis (91). GM130 is involved in nervous system diseases for its various physiological functions.

### Alzheimer's Disease

Alzheimer's disease (AD) dementia is a specific onset and course of disease in which age-related cognitive and functional decline is accompanied by a particular neuropathology. The initial stages of AD are characterized by the defective ability to encode and store new memories, followed by progressive changes in cognition and behavior (92). At present, the treatment strategy of AD mainly uses acetylcholinesterase inhibitors as cognitive enhancers and non-steroidal anti-inflammatory drugs, which can delay the occurrence and development of AD and alleviate cognitive dysfunction (93).

The accumulation of abnormally folded amyloid-β (Aβ) is causally associated with neurodegenerative processes in patients with AD (94). As a cleavage product of amyloid precursor protein (APP), Aβ peptide is involved in regulating neurite growth, cell adhesion, synaptogenesis, etc. as a cell surface receptor (95). The hippocampal tissues of transgenic AD mice expressing the APP Sweden mutation and presenilins 1 (PS1) deletion mutation were observed by fluorescence microscopy. The GA were scattered in fragments, contrary to the perinuclear ribbon organization of wild-type mice (96). In addition, since the earliest stages of disease development, GC fragmentation and dispersion has been observed in the neurons of patients with AD (86). At the ultrastructural level, the Golgi stack appears to be broken and of decreased diameter, and there are vesicles near the stacks (97). Losing the correct Golgi structure may change the correct speed and sequence of protein transport through Golgi membranes, which would change APP classification and processing, leading to the increased production of Aβ (96, 98, 99). However, the most important function of GM130 is to maintain the structure of GA.

In AD, the Cdk5 activity is aberrant, so deregulated Cdk5 might be involved in Golgi disassembly (100). The identification of Cdk5 phosphorylation site on GM130 showed that the deregulation of Cdk5 in AD might lead to GA fragmentation, whereas GM130 is a substrate of Cdk5 (100). At the beginning of the early prophase, GM130 is phosphorylated by Cdc2. It remains this state in metaphase and anaphase (27). The phosphorylation of Ser-25 disrupts the interaction between vesicle-docking protein p115 and GM130, resulting in GA fragmentation. GM130 is dephosphorylated at telophase, leading to the reassembly of Golgi (27). Cdk5 might act in a similar manner as Cdc2, due to its similar substrate specificity with Cdk5 (100).

### Parkinson's Disease

As the most common severe movement disorder, Parkinson's disease (PD) is age-dependent, affecting about 1% of adults over 60 years old (101). Idiopathic PD is related to risk factors such as age, family history, environmental chemicals, and pesticide exposure due to pathophysiological loss or degeneration of dopaminergic neurons in the midbrain substantia nigra and neuronal Lewy bodies development (102). PD patients typically present with shaking, resting tremor, rigidity, bradykinesia, stooping posture, slow movement, instable posture, and difficulty in walking and gait (102, 103). PD patients have difficulty in hand function and walking, as symptoms of the disease become more pronounced. They are prone to falls. Although the ultimate cause(s) of PD is (are) unknown (102), it is caused by loss of dopaminergic neurons (53).

The molecular mechanisms underlying selective dopaminergic neuronal degeneration remain unclear, although lots of reports have shown that genetic factors are involved in PD pathogenesis (104). It is characterized by the accumulation of α-synuclein (α-syn), a synaptic protein, existing in the form of amyloid fibrils in neurites and Lewy bodies of the nervous system (105). Lysosomal storage disorder (LSD) is the most common cause of pre-adult neurodegeneration, and the accumulation of storage vesicles in cells is considered as a feature of lysosomal storage diseases (90). Loss-of-function mutation of metabolic genes is an important risk factor for PD and other common neurodegenerative diseases (106). As shown in AD and PD, alterations in endolysosomal and/or macroautophagy pathways are closely related to neurodegeneration (107–109). A major obstacle in PD treatment is lacking identifiable therapies to reduce aggregation in human neuronal model systems (110). Lysosome dysfunction leads to α-syn accumulation and PD pathogenesis (111). Decreased expression of GM130 can alleviate abnormal lysosomal formation in HeLa cells lacking NAGLU, whereas overexpression of GM130 can lead to the formation of abnormal lysosomal with functional defects (18).

Genetic analysis has suggested that defective vesicle trafficking can also lead to PD (112, 113). The coiled-coil structure of GM130 and its combination with p115 can capture vesicles and participate in vesicle transport. P115, GM130, and Giantin complexes are responsible for mediating COPII vesicles to the cis surface of GA (53). Researches indicated that α-syn disrupts vesicle trafficking in the early secretory pathway (110, 114–116). COPII vesicle fusion with cis-Golgi requires rab1a-GM130 interactions (117). Mazzulli et al. (110) found that α-syn aggregation at the cell body led to abnormal association with GM130 and disrupted ER-Golgi localization of rab1a, an important mediator in vesicle transport, and then disrupts COPII vesicle fusion, leading to Golgi fragmentation.

Furthermore, *DJ-1* is a pathogenic gene in the autosomal recessive form of PARK7-linked early-onset PD, while *DJ-1* is co-located with GM130 and synaptic vesicle proteins, including rab3a and synaptophysin (104).

### Amyotrophic Lateral Sclerosis

ALS is a fatal idiopathic neurodegenerative disease of human motor system (118), characterized by degeneration of upper and lower motor neurons, resulting in muscle weakness and eventually paralysis (119). The initial symptoms are concentrated in random areas of the body (120). At onset, the pathological process of clinical manifestations is focal and distributed randomly throughout the nervous system (121). No definitive diagnostic test or biomarker for ALS exists at present, and neurologists have to rely on clinical diagnostic criteria (118). There can be many reasons for the same phenotypes, such as different genetic mutations. Thus, a variety of molecular mechanisms may cause ALS, which means the disease is a syndrome (122). There are two possible treatments for ALS that slow the progression of the disease, but patients are primarily treated with symptomatic therapies, including speech therapy for dysarthria and muscle relaxants for spasticity (119).

Alterations in GA can be detected in degenerating ALS motor neurons of cerebral motor cortex and spinal cord (88, 123). In ALS patients, the GA of motor neurons is fragmented, studied with an organelle-specific antiserum (124, 125). GA-fragmented motor neurons were moderately atrophied (126). In those cells, the number of discrete immunostained elements in organelles was more than twice that of normal neurons, and both the proportion of GA in cytoplasmic area and the size of each Golgi element decreased (126). The fragmentation of GA of motor neurons in ALS perhaps represents early change of organelle which is possibly involved in ALS pathogenesis (125), while GM130 helps to maintain the integrity of GA.

Among the specific gene mutations causing ALS, the most common is the mutation of superoxide dismutase 1 (SOD1), a powerful antioxidant enzyme protecting cells from the damage of superoxide radicals (127). The Golgi ribbon was observed to disintegrate into disconnected Golgi stacks, vesicles, and tubules in motor neurons of mutant SOD1 mice (128). Bellouze et al. (128) investigated the possible subcellular redistribution of Golgi tethers in these mice via biochemically dividing spinal cords into vesicles, membranes, and cytosol fractions. They found that GM130 was significantly redistributed, indicating that the expression of mutant SOD1 caused Golgi vesicle tethering defects due to GM130 redistribution. This might be conducive to developing new blood biomarkers for ALS.

### Microcephaly

The occipito-frontal head circumference of MCPH is below the third percentile, or 2 standard deviations (SD) or more lower than the mean for age, sex, and race (129, 130). The pathogenesis of MCPH is heterogeneous, ranging from genetic causes to environmental factors that can have an impact on developmental process influencing brain size (131, 132). Primary MCPH present at birth is a static developmental anomaly, while secondary MCPH develops postnatally and is a progressive neurodegenerative disease (133). Both primary and secondary MCPH could be acquired or genetic (134). Different causes and severity of MCPH may lead to different symptoms in children, including development retardation, intellectual disabilities, cerebral palsy, epilepsy, as well as vision and hearing disorders (135).

In 2016, Shamseldin et al. (21) suggested the important role of GM130 in human and zebrafish development. They found that a female patient with GM130 homozygous mutation had obvious MCPH, low muscle tone and growth retardation at 4 months of age, and infantile spasm at 6 months. All symptoms showed a trend of gradual aggravation with age. Magnetic resonance imaging showed non-specific brain volume reduction, delayed myelin sheath, and thinned myelin sheath. Electroencephalogram showed hypsarrhythmia. Muscle biopsy showed non-specific mild atrophy. The patient's clinical manifestations were speculated as the result of abnormal sorting or post-translational modification of proteins, since GM130 was not localized to the cis-Golgi (21). No possible pathogenic mutation was found in clinical triple exome sequencing. The whole exon sequencing results revealed that GM130 had 4 bp deletions, leading to early termination of protein translation. GM130-knockout zebrafish with the same mutant GM130 form showed similar symptoms to this patient: severe skeletal muscle development disorder and progressive MCPH.

Loss of the mitotic function of GM130 may be a cause of growth deficiency and MCPH (21). GM130 provides a long-sought molecular connection between cytoskeleton and GA, and thus its deficiency contributes to GA fragmentation and prevents normal mitosis (23, 26, 27). In order to promote the proper partitioning of mitotic cells, Golgi is decomposed by inhibiting vesicle fusion (67). The p115-mediated fusion of vesicle with Golgi membranes is reduced during mitosis, and the secretory pathway is down-regulated (136, 137). This triggers the formation of Golgi vesicles, which is required for the Golgi partitioning during cell division (138). The mitotic phosphorylation of GM130 on Ser-25 is believed to be responsible for the reduced binding of p115 to Golgi membranes (27, 136–138). Chang et al. (67) found that the binding of Importin-α and GM130 regulates Golgi disassembly, and thereby controls mitotic progression. The phosphorylation of GM130 also could promote the disintegration of GA during mitosis (23).

Moreover, many Mendelian diseases are caused by mutations in genes encoding GA components, and these are multisystem disorders resulting from perturbed posttranslational glycosylation of proteins in GA (139, 140), while GM130 is involved in providing a suitable glycosylation environment.

### Sepsis-Associated Encephalopathy

As a systemic inflammatory response, sepsis is life-threatening and common in patients with bacteremia (141, 142). SAE, a frequent sequela of sepsis, is a diffuse brain dysfunction without direct central nervous system (CNS) infection (141, 142). The patients present with acute mental state changes. SAE is associated with increased mortality and morbidity rate in septic patients (142, 143). When it occurs as a manifestation multiple organ dysfunctions, the mortality in SAE is estimated at 70% (142). Treatment of SAE is still limited to managing underlying infections (142, 144, 145).

Blood-brain barrier (BBB) is critical in the establishment and maintenance of brain (146). The disruption of BBB is implicated in SAE pathogenesis (147). The permeability of BBB is altered to regulate the substances transporting in blood and brain (148), thereby maintaining a state of homeostasis in the CNS (149). When BBB is damaged, its permeability increases. Endotoxin and inflammatory factors then enter the brain tissue, resulting in impaired or even loss of brain function (150). Endothelial Nitric Oxide Synthase (eNOS) regulates many key functions of vascular endothelial cells and plays an important role in maintaining the function of BBB (151). The normal structure and complete function of GA could effectively maintain the circulation and transport of eNOS in cells (152). GA is involved in regulating calcium ion release. Ca^2+^ release due to GA rupture may activate eNOS on the plasma membrane in a Ca^2+^/calmodulin-dependent manner and aggravate BBB injury (152). Moreover, GA is indispensable in maintaining the normal function of endothelial cells (ECs), tight junctions (TJ) protein, and astrocytes (152). Deng et al. (152) speculated that protecting GA might be a new therapy to protect BBB and treat nervous system diseases caused by BBB dysfunction.

Tight junctions are important parts of BBB (153, 154). Overactivation of Cdc42 leads to TJ degradation (155), whereas the appropriate level of Cdc42 activation promotes BBB integrity by assembling TJ proteins (156, 157). In the study of Baschieri et al. (11), Cdc42 appeared in a parallel cell pool, co-located with GM130. Loss of GM130 released RasGRF and inhibited Cdc42, leading to changes in cell polarity (11). Kodani et al. (65) demonstrated that GM130 regulates the activation of Golgi-localized Cdc42 via promoting the interaction between Golgi-localized Cdc42 and Golgi-localized Tuba.

Qiu et al. (158) conducted real-time polymerase chain reaction and Western blotting, aiming to explore the changes of tight junction protein, Cdc42, GM130, and mRNA expression in the brain of rat after intracerebral hemorrhage (ICH). They found that both the protein and mRNA levels of GM130 decreased significantly after ICH, and the structure of Golgi changed or even disintegrated, suggesting that GM130 may participate in the destruction of BBB through oxidative stress after ICH, which is related to the activation of apoptotic hydrolase induced by oxidative stress after ICH, thus hydrolyzing GM130.

### Ataxia

Cerebellum participates in motor control. Its damage leads to ataxia, a syndrome of incoordinate movement (159). Ataxia can be inherited, acquired, or sporadic (160). The causes of cerebellar ataxia are various, from infectious, immune mediated, to degenerative (161). Symptoms and signs are usually associated with the location of lesions in the cerebellum: lesions in the cerebellar hemisphere result in limb (appendicular) ataxia; lesions of the vermis lead to truncal and gait ataxia with limbs relatively spared; vestibular cerebellar lesions cause vertigo, disequilibrium, and gait ataxia (162). The treatments of ataxia include symptomatic and disease-modifying therapies (161). The sporadic adult-onset ataxia of unknown etiology still remains a diagnostic challenge (163). Few ataxias are completely treatable, but the promise of effective gene therapy and drug therapy is emerging.

Patients with several types of congenital disorders of glycosylation (CDG) caused by mutations in the genes that encode Golgi-associated proteins show ataxia (164). Liu et al. (20) reported that the targeted deletion of golgin GM130 resulted in a profound neurophenotype in mice: GM130-knockout mice showed severe ataxia, developmental delay, and postnatal death. They indicated that the selective GM130 deletion in neurons resulted in GA fragmentation and positioning defects, impaired secretory transport, as well as dendrite atrophy in Purkinje cells (20). Cellular defects are characterized by decreased cerebellar size and Purkinje cell number, causing ataxia (20).

Expansion of polyglutamine repeats to 32 or longer leads to SCA2 (89). In this disease, mutant ataxin-2 mainly contributes to the neurodegeneration of Purkinje neurons and selected neurons in the brain stem, leading to ataxia and death (165–167). Research showed that ataxin-2, the product of *SCA2* gene, was predominantly located in GA, and suggested that the mutant ataxin-2-mediated cell death was related to GC stability (89). Key to maintaining GA is a set of Golgi tethering proteins, connecting Golgi stacks to a ribbon (30, 168, 169). GA has a characteristic structure, consisting of one or more stacks of cisternae, laterally connected to form Golgi ribbon in vertebrate cells (170, 171). GM130 contributes to GA morphology. Experimental depletion of GM130 resulted in the loss of the ribbon architecture into stacks (172). Golgi assembly was inhibited by disrupting GM130-p115 complexes with competing peptides or antibodies, or by the expression of GM130 mutants (23, 173) (Table 2).

**Table 2:** The role of GM130 in nervous system related diseases.

**References**	**Diseases**	**Cause**	**The role of GM130**
Marra et al. (30), Mitchell et al. (31)	AD	Fragmentation of GA	GM130 maintains the structure of GA
Hunn et al. (112), Martin et al. (113), Alvarez et al. (53)	PD	Defective vesicular transport	The coiled-coil structure of GM130 and its combination with p115 participate in vesicle transport
Wallis et al. (174)	ALS	Fragmented GA of motor neurons	The alteration of GM130 labeling suggests the fragmentation of GA
Shamseldin et al. (21)	MCPH	Fragmentation of GA and abnormal mitosis of cells	This may be due to the loss of mitotic function of GM130
Deng et al. (152)	SAE	The disruption of BBB	GA protects BBB, while GM130 maintains the structure of GA
Liu et al. (20)	Ataxia	Related to the stability of the GC	Selective deletion of GM130 in neurons leads to the fragmentation and defective positioning of GA

*GA, Golgi apparatus; BBB, blood-brain barrier; GC, Golgi complex; AD, Alzheimer's disease; PD, Parkinson's disease; ALS, amyotrophic lateral sclerosis; MCPH, microcephaly; SAE, sepsis-associated encephalopathy*.

## Conclusions and Prospects

The biological function of GM130 involves all aspects of life process. At present, its research in nervous system diseases is still very limited. A number of studies have confirmed that GM130 is critical for the maintenance of the typical ribbon structure of GA in mammalian cells. In many neurodegenerative diseases, the GA is fragmented. Therefore, targeted therapies designed to protect or restore GA may be a treatment for CNS diseases in the future. GM130 plays an important role in material transport, cell mitosis, migration and polarity, glycosylation, as well as lysosome formation. This also creates potential for the development of new drugs targeting GM130 and the treatment of various diseases. Due to the diversity of physiological functions of GM130, there are great limitations in study at the individual level. At present, the research in this field is still in the early stage and has not formed a systematic research system. Further studies on animal models are expected to be carried out on the basis of existing studies at the cellular level. Thus, the results of the study on the physiological function of GM130 at the individual level could be applied to the detection and treatment of corresponding diseases and the development of specific targeted therapy.

## Author Contributions

BH drafted manuscript and prepared tables and figures. XL edited and revised manuscript. XZ edited figures. BH, XL, and XZ approved the final version of manuscript. All authors contributed to the article and approved the submitted version.

## Funding

This work was supported by the National Natural Science Foundation of China: 82071353 (to XL), the National Key Research and Development Program of China: 2017YFA 0104201 (to XL), and Key Research and Development Projects of Sichuan Province in China: 2021YFS0029 (to XL).

## Conflict of Interest

The authors declare that the research was conducted in the absence of any commercial or financial relationships that could be construed as a potential conflict of interest.

## Publisher's Note

All claims expressed in this article are solely those of the authors and do not necessarily represent those of their affiliated organizations, or those of the publisher, the editors and the reviewers. Any product that may be evaluated in this article, or claim that may be made by its manufacturer, is not guaranteed or endorsed by the publisher.
